# Development of new TB regimens: Harmonizing trial design, product registration requirements, and public health guidance

**DOI:** 10.1371/journal.pmed.1002915

**Published:** 2019-09-06

**Authors:** Christian Lienhardt, Andrew A. Vernon, Marco Cavaleri, Sumati Nambiar, Payam Nahid

**Affiliations:** 1 Unité Mixte Internationale TransVIHMI, UMI 233 IRD–U1175 INSERM—Université de Montpellier, Institut de Recherche pour le Développement (IRD), Montpellier, France; 2 Division of TB Elimination, National Center for HIV, Viral Hepatitis, STD and TB Prevention, Centers for Disease Control and Prevention, Atlanta, Georgia, United States of America; 3 Office of Anti-infective and Vaccines, Human Medicines Evaluation, European Medicines Agency, Amsterdam, the Netherlands; 4 Division of Anti-Infective Products, Center for Drug Evaluation and Research, US Food and Drug Administration, Silver Spring, Maryland, United States of America; 5 Division of Pulmonary and Critical Care Medicine, Center for Tuberculosis, University of California, San Francisco, San Francisco, California, United States of America

## Abstract

Christian Lienhardt and colleagues discuss the importance of communication and coordination between regulators, researchers, and policy makers to ensure tuberculosis trials provide high-quality evidence for policy decisions.

**Summary points**

- Regulatory approval of new tuberculosis (TB) drugs can be based on data from trial(s) using a surrogate endpoint of treatment efficacy under an accelerated or conditional procedure. In such circumstances, policy makers and TB programs can be hampered in their ability to make recommendations on the optimal use of the drug(s), and consequently, the uptake by national or international public health institutions of such recommendations can be limited.
- Based on the essential need to produce high-quality evidence for policy decisions, this paper reflects on specific methodological issues in clinical trial design that need to be addressed to improve compliance with clinical, regulatory, and public health requirements.
- Established mechanisms for communication between drug developers and regulators already exist; however, equal engagement with policy makers is also essential for the optimal selection of trial designs, endpoints, and markers of treatment outcome and for giving consideration to public health and program aspects.
- The next generation of TB trials should better reconcile the research agenda with the need for global policies on access to TB medicines. Policy decision-makers should establish formal mechanisms for iterative feedback on regimen-development pathways. In this paper, we provide examples of how the need for interactions between regulators, trialists, and policy decision-makers can be addressed.

 

## Introduction

Under the paradigm of adding a new drug to a regimen or substituting single drugs in a regimen one at a time, it would take 15–20 years to develop an entirely new tuberculosis (TB) regimen comprising three to four new drugs [1]. As has been noted in the papers of this Special Collection on Advances in Clinical Trial Design for Development of New TB Treatments [2–4], the major challenges in the development of new TB treatments include the long developmental pathway to identify best regimens, the uncertainties around the correlation between the treatment effect and existing surrogate endpoints, and uncertainties around the predictive quantitative relationships between Phase II and Phase III trial outcomes. Beyond measures of efficacy, the development of shorter, simpler regimens combining new and existing drugs also requires detailed information on their respective safety and toxicity, their potential for drug–drug interactions, their propensity for development of drug resistance while on therapy, and their use in specific patient populations such as persons infected with human immunodeficiency virus (HIV), pregnant women, and children [5].

Over the last decade, a series of clinical trials have been carried out to assess the safety and efficacy of new or repurposed drugs for the treatment of TB [6]. Although in some of these trials the endpoints were selected to address regulatory requirements, such endpoints were not always optimal to draw inferences for policy-recommending institutions, such as the World Health Organization (WHO), that provide guidance on the optimal use of these drugs in combination treatment regimens [2]. Ideally, clinical trials should provide results that are as meaningful as possible for clinical, regulatory, and programmatic perspectives. In situations when the regulatory approvals are conditional, based on surrogacy or on preliminary limited clinical data sets, the question is posed as to what extent policy makers can suitably generate comprehensive recommendations on the optimal use of the drug(s) in combination regimens. What needs to be considered in the design of a clinical trial to have relevance across regulatory and programmatic requirements? The design and choice of specific endpoints in trials of new TB drugs and regimens have implications for the development of guidelines and their adoption by national or international public health institutions. Starting from the need to produce evidence of high quality, this paper reflects on study designs and endpoints that respond best to the combined clinical, regulatory, and public health requirements.

## The regulatory needs

In principle, regulatory authorities overseeing drug development have the primary responsibility of ensuring that the quality, efficacy, and safety of marketed medicinal products are adequate, conforming to currently defined standards. A key role of the regulatory authorities is to determine whether there is a positive benefit–risk balance to support use of the drug for the proposed indication and patient population.

Regulators also continue to reevaluate the benefit–risk balance after approval through pharmacovigilance activities and postmarketing studies. New data that emerge in the postapproval phase are taken into consideration in reassessing the benefit–risk balance, and information is communicated in product labeling as appropriate. Regulators, however, are not expected to consider cost-effectiveness or to perform in-depth evaluations of comparative effectiveness in assessing benefit and risk or for defining treatment policies. This role lies, rather, within the scope of public health recommending bodies, and, even if at times there seems to be some overlap, it is important to recognize and understand the implications of this distinction.

Some regulatory agencies have mechanisms for accelerated reviews and early approval of new drugs that address unmet needs according to specified criteria—e.g., the conditional marketing authorization pathway in the European Union where the benefit–risk balance of the new drug is such that immediate availability justifies acceptance of less comprehensive data than normally required [7, 8]. In the United States, the accelerated approval pathway allows for the approval of a product for a serious disease with an unmet need based on a surrogate or an intermediate clinical endpoint that is reasonably likely to predict clinical benefit [9]. The accelerated approval pathway has been used primarily in conditions in which the disease course is long and an extended period of time would be required to measure the intended clinical benefit of a drug. The implication is that, while awaiting further data to be generated postapproval, there may be limited data to support policy recommendations at this stage.

Development of new TB drugs and regimens is a good example of a scenario in which regulators need to establish that a drug submitted for licensure is safe and effective for the proposed use, whereas recommending bodies need to define how to use the drug optimally within a regimen in a way that addresses the public health need. Often, demonstrating the safety and effectiveness of a drug is the first step. Although a single clinical study cannot answer all research questions at once, it is still worth exploring clinical study designs that maximize the chance of gathering evidence that is informative both for assessing the benefit–risk of individual drugs and for determining their optimal use in the context of TB regimens. In view of the shift in focus toward the development of new treatment regimens, the European Medicine Agency (EMA) has proactively issued updated guidance to developers to address such scenarios [10]. In July 2017, the US Food and Drug Administration (FDA) held a public workshop regarding scientific and clinical trial design considerations for development of new TB drug regimens [11]. Of note, the FDA and EMA work collaboratively to provide advice to pharmaceutical sponsors or investigators on various aspects of the clinical trial design and to ensure that, whenever feasible, the same development program addresses the regulatory requirements of these agencies (for instance, the FDA pre–investigational new drug (IND) consultative process allows facilitated early communications between the FDA and potential drug sponsors or investigators [12]).

## The public health needs

Countries, technical agencies, donors, and other TB stakeholders, routinely seek guidance and advice from WHO on optimal disease management practices to be adopted based on the evidence available. Over the last decade, WHO has published a series of normative guidance documents for the diagnosis and treatment of all forms of TB, with a particular focus on the needs of low- and middle-income countries [13]. In 2007, WHO adopted a procedure to guarantee that guidelines are based on the best available evidence and meet the highest international standards. Using the *Grading of Recommendations Assessment*, *Development*, *and Evaluation* (GRADE) framework, which relies on the use of systematic reviews and meta-analyses, the findings of these reviews are then considered in the context of implementation and feasibility issues of stakeholder countries [14, 15]. The GRADE framework provides an explicit and transparent approach to assess the level of certainty in the evidence across relevant studies and outcomes and to translate that evidence to recommendations. This framework incorporates multiple processes to minimize bias and optimize usability and requires rigor, fairness, and transparency in all judgments and decision-making.

To formulate evidence-based recommendations, four key aspects are taken into account: (1) the respective magnitude of benefits and harm conferred by the intervention under evaluation; (2) the consideration of resource use, feasibility, acceptability, and equity; (3) the certainty (“quality”) of evidence; and (4) patients’ values and preferences. Based on this assessment, the proposed recommendation is qualified as “strong” or “conditional” (i.e., “weak”), reflecting the extent to which one can, across the range of patients for whom the recommendation is intended, be certain in the evidence that the desirable effects of the given intervention outweigh the undesirable effects. The assessment of each of the above aspects leads, understandably, to the consideration of a number of nuances when moving from clinical trial results to public health policy making. As a result, the final qualification of the recommendation ultimately has implications for the way policy makers, clinicians, and patients interpret and adopt the guidance, as shown in Table 1.

**Table 1:** Implications of GRADE recommendations.

Target population	Strong recommendation	Conditional/weak recommendation
Policy makers	The recommendation can be adapted as a policy in most situations	There is a need for substantial debate and involvement of stakeholders
Patient	Most people in this situation would want the recommended course of action, and only a small proportion would not	The majority of people in this situation would want the recommended course of action, but many would not
Clinician	Most patients should receive the recommended course of action	Be more prepared to help patients to make a decision that is consistent with their own values/decision aids and shared decision-making

Abbreviation: GRADE, *Grading of Recommendations Assessment*, *Development*, *and Evaluation*

Recent developments highlight how trial results that are used as the basis for regulatory approval may allow only conditional recommendations for policy making due to the use of surrogate endpoints and limited data on patient- and population-relevant outcomes. As an example, the accelerated approval of bedaquiline by the US FDA in December 2012, based on the surrogate endpoint of sputum culture conversion at 6 months, allowed the drug to be readily used in the treatment of multidrug-resistant (MDR)-TB under certain conditions in the field [16]. However, the data gathered from the pivotal Phase II trial appeared inadequate for policy decision-making because of the absence of information on the outcomes of interest (nonrelapsing cure); further, the selected design did not provide information on the optimal use of the drug in combination with others or whether the addition of the drug would allow any modification in treatment duration. Finally, there was an excess of deaths in the experimental arm, the significance of which was uncertain given the small sample sizes and lack of long-term follow-up. These limitations in the available evidence at the time of regulatory review led to the adoption of a conditional recommendation that had implications in terms of wider scale-up of the intervention. Thus, for bedaquiline, results of the pivotal Phase II trial, in addition to relevant safety data, were adequate for obtaining regulatory approval but appeared insufficient for wider policy recommendations [17], thus calling for postlicensure evidence generation. The yield of a large body of observational data obtained over a subsequent period, associated with large individual-patient data meta-analyses, allowed WHO to update its recommendations for MDR-TB treatment in December 2018 [18], with significant changes in the assessment of the quality of evidence. As a result, bedaquiline is now strongly recommended for use in the treatment of MDR-TB, based on moderate-quality evidence—showing the importance of collecting additional data to complement early trial results. It should be noted that, at the time, the standard of care for rifampicin-resistant (RR)-TB treatment had low efficacy and high toxicity and was based on observational evidence. Though these conditions are now changing, a similar situation may present itself again in the future. Therefore, the experience with bedaquiline raises the question of whether specific trial features and designs can be used to produce endpoints with value for both the regulator and the policy maker. It is with this objective in mind that the Task Force on New Drug Policy Development established by WHO in 2012 worked together with drug developers, regulators, scientists, and program managers to define the policy needs and produce relevant documents [19].

## Methodological issues: How to fit both regulatory and programmatic decision-making needs

Could outcome definitions in clinical trials be redesigned to satisfy both regulatory and programmatic decision-making needs? We argue that this is feasible, and WHO Technical Consultation on Advances in Clinical Trials Design for TB Treatment Regimen proposed features and designs that could address this need in greater detail and that are described in relevant papers of this Collection [2, 20].

Regulatory agencies rightfully seek to use conservative approaches to endpoint evaluation, relying upon the protection from bias provided by randomization. For certain diseases, including MDR-TB, the expedited approval pathway can be used based on a surrogate or an intermediate clinical endpoint that is reasonably likely to predict a clinical benefit. These endpoints, however, are not fit-for-purpose for programmatic and policy needs. Whereas intensive efforts are underway to identify improved intermediate surrogate markers of treatment outcome with the ability to measure and describe accurately the effect an experimental regimen will likely have on achieving nonrelapsing cure [21, 22], no marker has yet been identified that fully serves the needs of TB investigators and regulators, let alone policy makers [23]. The desire for an equivalent to the viral load in HIV and viral hepatitis trials has been often voiced but not yet attained, and current efforts are directed toward identification of markers that might reliably predict efficacy. In addition, combination of bacterial (e.g., minimum inhibitory concentration [MIC]) and host (e.g., pharmacokinetic characteristics, adherence, and perhaps genetic or other features) factors would be of value in dose selection and for predicting outcome [24, 25]. Relevant surrogate markers providing highly reliable estimates of treatment outcome, once realized, could provide sufficient evidence for guideline development beyond market approval [4], but until then, the TB therapeutics field has to look to novel trial designs, long-term endpoint definitions, and other trial features as a means to generating data pertinent to policy decisions [3].

The “composite” clinical trial endpoint (comprising multiple events such as a combination of failure, relapse, and death) has been used as a mechanism to capture multiple serious outcomes of interest with a programmatic perspective, often allowing for smaller sample sizes. The use of composite endpoints, however, poses some problems, the most significant being that respective endpoints are of differing individual and public health value (i.e., death is always a worse outcome than any other). Further, there are often varying levels of certainty around different endpoints (for example, cause of death is often uncertain in trials performed in low-resource settings). The choice of the components of a composite endpoint should be made carefully: because the occurrence of any one of the individual components is considered to be an endpoint event, each of the components is of equal importance in the analysis of the composite [26]. For these reasons, when composite outcomes are used, it is essential that information on all their components be collected in such a way that they can be disaggregated and individually reported. As an illustration, endpoints of currently conducted Phase II and Phase III trials of TB drugs or regimens are shown in Table 2.

**Table 2:** Recent and current Phase II and Phase III trials of new TB drugs or regimens, with their respective endpoints. (Trial names shown with a blue background involve DS TB; those with a gray background involve DR-TB).

Phase II trials				
Trial name (registration no.)	Phase	Sample size	Study groups; +/− dates; locations; sponsor	Primary efficacy endpoint (per online registration)
APT (NCT02256696)	2B	183	2 months Pret + RHZ daily and 1 month, Pret + RH daily, or 2 months Pret + Rifabutin + H + Z daily, and 1 month Pret + Rifabutin + H daily, versus 2 months HRZE daily, and 1 month HR daily Opened April 2015 (paused October 2016–May 2017), results expected 2020. John Hopkins University, University of Cape Town Lung Institute	• Time to SCC in liquid medium (≤12 weeks); • Grade ≥ 3 AEs
HIGHRIF-1 Extension	2	30 HIV–adult	EBA safety, tolerability, PK study Opened September 2017, results mid-2018; PanACEA	• Rate and severity of AE with increasing doses of rifampicin up to 50 mg/kg given as single drug or with HEZ
Janssen C211 (NCT02354014)	2	60 (ped)	PK, safety, dose-range 6 months Bdq (daily for 2 weeks, then 3 times a week) plus OBR, single-arm study Opened May 2016, results March 2021; India, Philippines, Russia, South Africa; Janssen	•Number with AE or SAE; • PK parameters
NC-005 (NCT02193776)	2B	60	Serial sputum culture counts: 8 weeks Bdq (200 mg daily) + Pret (200 mg daily) + M + Z, single-arm study with long follow-up Opened November 2014, preliminary findings presented at CROI, 2017 (#724LB), final results expected 2019; TB Alliance	• Bactericidal activity as determined by the rate of change in time to sputum culture positivity or by TTP in MGIT
OPTI-Q (NCT01918397)	2	100	6 months Lfx (14, 17, or 20 mg/kg/day) plus OBR versus 6 months Lfx (11 mg/kg/day) plus OBR Opened January 2015, results expected end 2019; South Africa, Peru. NIAID, Boston University, CDC TBTC	• Time to SCC from positive to negative for *Mycobacterium tuberculosis* growth on solid medium
Stage 2 STEP	2C	600 HIV− adults	4 months R (high dose)+H+Z+E, 4 months R (high dose)+H+Z (high dose)+E, 3 months sutezolid (optimal dose)+Bdq+Del+M versus 2HRZE/4HR. Adaptive trial design, examining new treatment backbones; PanACEA	• This trial will be informed by findings of a Phase II study to be carried out in drug-sensitive TB patients, the SUDOCU trial (NCT0395966). This is a dose range study of sutezolid (0 mg qd, 600 mg qd, 1200 mg qd, 600 mg bid, or 800 mg bid), all for 3 months combined with 3 months of daily Bdq, Del and M. N = 75.
Phase II/III trials				
Trial name (registration no.)	Phase	Sample size	Study groups; +/− dates; locations; sponsor	Primary efficacy endpoint (per online registration)
NC-008 SimpliciTB (DS) (NCT03338621)	2C/3	300	4 months Bdq + Pret + M + Z versus standard 6-month therapy Opened August 2018, results expected 2022; TB Alliance	•Time to culture negative over 8 weeks (secondary outcome = bacteriologic failure/relapse, or clinical failure, at 52 and 104 weeks from start of therapy)
NC-008 SimpliciTB (DR) (NCT03338621)	2C/3	150	4 months Bdq + Pret + M + Z, single-arm study Opened August 2018, results expected March 2022; TB Alliance	•Time to culture negative over 8 weeks (secondary outcome = bacteriologic failure/relapse, or clinical failure, at 52 and 104 weeks from start of therapy)
NExT (NCT02454205)	2/3	300	6–9 months Bdq + Lzd + Lfx + Z, and either high-dose H or ethionamide or terizidone daily (all oral) versus 6–8 months kanamycin + M + Z + ethionamide + terizidone daily, then 16–18 months MZEthTer Opened October 2015, results expected 2019; University of Cape Town	• Treatment success, defined as the sum of cured and treatment-completed cases (standard arm), without relapse, reinfection, or death during the 15–18 month follow-up period (test arm)
TB-PRACTECAL (NCT02589782)	2/3	630	6 months Bdq + Pret + M + Lzd daily, or 6 months Bdq + Pret + Lzd + Cfz daily, or 6 months Bdq + Pret + Lzd daily (all oral) versus local regimen Opened January 2017, results March 2021; Belarus, South Africa, Uzbekistan; MSF	Percent with culture conversion in liquid media at 8 weeks; percent unfavorable at 72 weeks (failure, death, recurrence, loss to follow-up)
TRUNCATE-TB (NCT03474198)	2/3	900	2 months various new regimens versus standard 6 months; regimens including H + R35 + Z + E + Lzd, H + R35 + Z + E + Cfz, H + Z + Rpt + Lzd + Lfx, H + Z + E + Lzd + Bdq Opened late 2017, results expected 2021; MAMS adaptive trial design. Thailand, Indonesia, Philippines, Singapore; BMRC, NUS	• Unsatisfactory clinical outcome at week 96 after randomization (active TB, TB treatment, or death)
MDR-END (NCT02619994)	2	238	9 or 12 months Del + Lfx (750 or 1,000 mg) + Lzd (600 mg daily for 2 months, 300 mg daily thereafter) + Z, versus local regimen Opened January 2016, results December 2019; Korea	• Treatment success 24 months after start of treatment (both “cured” and “treatment completed”)
Phase III trials				
Trial name (registration no.)	Phase	Sample size	Study groups; +/− dates; locations; sponsor	Primary efficacy endpoint (per online registration)
endTB (NCT02754765)	3	324	9 months Bdq + Lzd + M + Z daily, 9 months Bdq + Lzd + Cfz + Lfx + Z daily, 9 months Bdq + Lzd + Del + Lfx + Z, 9 months Del + Lzd + Cfz + Lfx + Z, or 9 months Del + Cfz + M + Z, versus local regimen Opened December 2016, results September 2020; Georgia, Kazakhstan, Kyrgyzstan, Lesotho, Peru; MSF, Partners in Health	• Proportion favorable at week 73 (not unfavorable, and culture negative at week 65–73, or earlier negative culture and no other evidence of unfavorable) • In addition, a companion phase 3 trial will be launched in drug-resistant TB patients, the “end TB-Q” trial (NCT03896685). This trial compares 6 months or 10 months of daily Bdq, Del, Lzd and Cfz versus WHO standard of care in DR patients with fluoroquinolone resistance.
Otsuka Trial 213 (NCT01424670)	3	511	2 months Del (100 mg twice daily) and 4 months Del (200 mg daily) plus OBR versus 6 months placebo plus OBR Opened September 2011, completed June 2016, preliminary findings presented at IUATLD October 2017, results published 2019; Otsuka	• Time to SCC, i.e., distribution of the time to SCC during the 6 months of study drug treatment
NC-006 STAND-DS (NCT02342886)	3	271 (orig 1,200)	4 months Pret (100 mg twice daily or 200 mg once daily) + M + Z daily, or 6 months Pret (100 mg twice daily) + M + Z daily, or 6 months Pret (200 mg once daily) + M + Z daily, versus standard 6-month therapy Opened February 2015, paused October 2016–May 2017; accrual not resumed; TB Alliance	•Incidence of combined bacteriologic failure or relapse, or clinical failure, at 12 months from start of therapy
NC-006 STAND-DR (NCT02342886)	3	13 (orig 300)	6 months Pret (200 mg) + M + Z daily, single-arm study Opened February 2015, paused October 2016–May 2017, accrual not resumed; TB Alliance	•Incidence of combined bacteriologic failure or relapse, or clinical failure, at 12 months from start of therapy
NiX-TB (NCT02333799)	3	109 (orig 300)	6 months Bdq (200 mg daily for 2 weeks and then 200 mg three times weekly) + Pret (200 mg daily) + Lzd (600 mg twice daily), single-arm study Opened March 2015, preliminary findings presented at CROI, 2017, accrual closed November 2017, with opening of NC-007 ZeNiX trial; TB Alliance	• Incidence of bacteriologic failure or relapse or clinical failure through follow-up until 6 months after the end of (6–9 months) treatment
NC-007 ZeNiX (NCT03086486)	3	180	2 or 6 months Lzd (600 or 1,200 mg daily, double-blind) + Bdq (200 mg daily for 2 weeks, then 100 mg daily) + Pret (200mg daily) Opened November, 2017, results January, 2021; TB Alliance	•Incidence of bacteriologic failure or relapse or clinical failure through follow-up until 26 weeks after the end of treatment; culture conversion requires at least two consecutive culture negative/positive samples at least 7 days apart
RIFASHORT (NCT02581527)	3	800	2 months H + R (1,200 or 1,800 mg) + Z + E daily and 2 months H + R (1,200 or 1,800 mg) daily, versus standard 6-month therapy Opened February, 2017, results expected January, 2020; St George’s London, INTERTB	•Combined rate of failure and relapse 12 months after end of treatment in mITT • Grade 3–4 AEs
SHINE (ISRCTN63579542)	3	1,200 (ped minimal disease)	2 months H + R (600 mg) + Z + (in some) E daily, and Z, and (in some) E daily, and 2 months H + R (600 mg) daily versus standard 6-month therapy Opened third quarter of 2016, results 2020; treatment-shortening strategy trial for children with minimal TB; India, Uganda, South Africa, Zambia; BMRC	• Unfavorable outcome (failure, relapse, death) • Grade 3–4 AEs
STREAM Stage-1 (ISRCTN78372190)	3	424	4 months daily M + Cfz + Z + E + high-dose H + kanamycin (daily for 3 months and then 3 times per week) + prothionamide, and 5 months of M + Cfz + Z + E daily, versus local standard Opened 2012, closed to accrual June 2015, preliminary findings presented at IUATLD October 2017, results early 2019; IUATLD, MRC, USAID	• Proportion of patients with a favorable outcome 132 weeks after randomization having not previously had an unfavorable outcome or been retreated
STREAM Stage-2 (NCT02409290, ISRCTN18148631)	3	1,155	9 months M + Cfz + E + Z daily, with initial 2 months of high-dose H + kanamycin + prothionamide daily, or 9 months Bdq + Cfz + E + Lfx + Z daily, with initial 2 months high-dose H + prothionamide daily (all oral), or 6 months Bdq + Cfz + Lfx + Z daily with initial 2 months high-dose H and kanamycin versus 20–24 month local regimen Opened April 2016, results expected April 2021; IUATLD, MRC, USAID, TB Alliance	• Proportion of patients with a favorable outcome at week 76 (noninferiority margin 10%)
TBTC 31/A5349 (NCT02410772)	3	2,500	2 months H + Rpt (1,200 mg) + Z + E daily, and 2 months H + Rpt (1,200 mg) daily, or 2 months H + Rpt (1,200 mg) + Z + M daily, and 2 months H + Rpt (1,200 mg) + M daily versus standard 6-month therapy Opened January 2016; results 2020; substudies include interactions of Rpt and efavirenz, intensive PK and pharmacodynamics of Rpt, and sputum biomarkers to predict outcomes; CDC TBTC, ACTG	•TB disease-free survival at 12 months after assignment •Proportion of participants with grade 3–5 AEs during treatment

Adapted from Tiberi and colleagues [6].

Abbreviations: AE, adverse event; Bdq, bedaquiline; BMRC, British Medical Research Council; Cfz, clofazimine; CDC, Centers for Disease Control; CROI, Conference on Retroviruses and Opportunistic Infections; Del, delamanid; DS, drug-sensitive; DR, drug-resistant; E, ethambutol; EBA, early bactericidal activity; H, isoniazid; HIV, human immunodeficiency virus; IUATLD, International Union Against Tuberculosis and Lung Diseases; Lzd, linezolid; Lfx, levofloxacin; MGIT, mycobacterial growth in-tube; MSF, Médecins Sans Frontiers; M, moxifloxacin; mITT, modified intent-to-treat; NCT, identifying registration number on www.ClinicalTrials.gov; NIAID, National Institute of Allergy and Infectious Diseases; NUS, National University of Singapore; OBR, optimized background regimen; orig, originally; ped, pediatric; PK, pharmacokinetics; Pret, pretomanid; R, rifampin 10 mg/kg; R35, rifampin at 35 mg/kg; Rpt, rifapentine; SCC, sputum culture conversion; TB, tuberculosis; TBTC, TB Trials Consortium; TTP, time to positivity; USAID, US Development Aid Agency; Z, pyrazinamide.

Noninferiority (NI) design has become the design of choice in most Phase II and Phase III trials of new TB drugs and regimens over the last decade, either because of the high efficacy of the control regimens (as in drug-susceptible TB) or because of the interest in shortening treatment (as in the case of DR-TB). NI trial designs, however, pose a number of methodological questions, particularly in terms of analysis [27]. In NI trial designs, different analysis populations are of interest—the effect in all randomized patients and the effect in those who can adhere to treatment, which have historically been estimated using the intention-to-treat (ITT) and the per protocol (PP) populations, respectively [28]. The ITT principle allows virtually all patients to contribute information to the primary trial analysis. In this approach, all randomized patients are included in the analysis of results, and favorable status is assigned only to those patients whose favorable outcome is documented; all others are deemed unfavorable or nonassessable (including those lost to follow-up, those whose therapy is altered, those who die or withdraw early, etc.). The PP population, conversely, is composed of those randomized and otherwise eligible participants who complete the trial without significant deviation from the intended trial behavior; in particular, such participants typically satisfy minimal requirements for adherence to the trial interventions. Analysis with each of these two populations should lead to similar conclusions for a robust interpretation [29]. The ICH E9 Guideline further specifies that “any differences between them can be the subject of explicit discussion and interpretation" [30]. This concern arises in part from the recognition that adherent participants differ in unknown ways from those who are not adherent, as they may have more favorable outcomes, no matter what their randomized therapy [31]. The analyses of these trials are most robust when there is a high level of adherence, as inadequate therapy in all trial arms may lead to equally poor performance across arms and nonadherers are imputed as treatment failures in the analysis of all randomized patients, risking creating a false conclusion of NI. Consequently, it is extremely important that trial protocols encourage a high level of adherence.

Finally, the generalizability of findings from preapproval clinical trials to the different populations and areas of interest to policy makers is also a significant concern. Some populations may be underrepresented in clinical trials conducted for approvals (e.g., children, elderly people, pregnant women, persons with advanced comorbid illness), whereas others are excluded for reasons of feasibility (e.g., those living far away from a clinic or deemed unreliable for follow-up). Significant problems have arisen from the assumption of generalizability [32]. When a successful trial establishes the efficacy of a new agent or regimen, efforts are then needed to expand exploration of the regimens in broader populations, or through additional pragmatic trials, such as the endTB trial [33]. The need for such trials is unlikely to be addressed through any innovations in design, but the rationale for excluding special populations even from early and middle phases of development is currently being revisited in the TB therapeutics field [2, 3, 20].

## The link between registration and public health recommendations: Implications for national TB programs and the way forward

For TB program managers and policy makers at the country level, the successful registration of a candidate drug is only one component of the decision-making process around adoption and use. Feasibility, acceptability, resource use, equity, and quality of life are also considered when formulating public health recommendations, and these rely on qualitative data that need to be collected in parallel to quantitative assessment of evidence.

WHO guidelines are key for the development of national policies for the care of TB patients. However, when reliable data are lacking, recommendations are predominantly based on low or very low certainty in the evidence, which creates challenges for the potential rapid adoption, successful implementation, and subsequent uptake of the new therapies—as has been the case with the treatment of DR-TB [34, 35]. Moreover, recommendations, even if based on low or very low certainty in the evidence, will often create the perception of a new “standard of care” that subsequently complicates the ability to fund and conduct pragmatic trials that would address the uncertainty left by the lack of data. Policy makers, donors, and ethical review bodies should be aware that significant uncertainty persists when recommendations based on very low or low certainty are adopted and that further research is essential to test the merits of the new standard of care proposed. Such additional research can generate postlicensure data that are important for the update of policies, as in the case of the recent WHO DR-TB treatment guidelines [18, 36] (Table 3).

**Table 3:** The interplay between trials and guidelines: Review of the proposals arising from WHO Technical Consultation on Advances in clinical trial design for development of new TB treatments (adapted from WHO [20]).

Issue	Expert consensus	To be explored	Research gaps
What clinical trial outcomes are required to inform regulatory and programmatic decision-making and need to be prioritized for prospective implementation in novel trial designs?	A single clinical trial cannot address all relevant regulatory and policy/public health questions. Explanatory trials, novel adaptive trials, pivotal trials for licensure need to be followed up with pragmatic trials to understand the optimal use of new drugs and regimens.	Consider postauthorization studies to answer some of the questions that cannot be addressed in the registrational trial(s) to help bridge gaps in knowledge.	Treatment success outcomes in recent trials of MDR-TB were much higher than that reported in prior trials and across program settings. Further research is needed to better understand the performance of the standard of care for rifampicin-susceptible and rifampicin-resistant TB in various conditions and settings to aid in the design of future studies.
How can current/novel clinical trial endpoints that are intended to support regulatory decisions be subsequently translated to support programmatic implementation?		Operational research can help to translate clinical trial outcomes into WHO guidance and add evidence for better programmatic implementation. Often, patients enrolled in trials are not reflective of the general population; consider ways to make trial population more reflective of the population of patients who will be receiving treatment in real life. Also consider pragmatic studies for better evidence on programmatic implementation.	
Should the assessment of clinical trial outcomes be updated for harmonization across regulatory and programmatic objectives, and if yes, how?	Communication between drug/regimen developers, regulators, and recommendation bodies is essential and should be encouraged and facilitated as early as possible at design stages.	Approaches to collecting clinical outcomes data that can potentially address assessment of safety and efficacy of the product and answer questions that are important from a programmatic perspective should address the following: • secondary/exploratory analyses are an option—but caution in overinterpreting the data • sample size implications if multiple primary analyses considered • importance of prespecifying analyses; consistent definitions across different trials are needed; limitations of using surrogate endpoints (e.g., 2-month culture conversion) for development of guidelines.	
How to ensure that trial data at the individual-patient level can be pooled for enhanced meta-analysis when reviewing evidence for policy making by WHO and other professional bodies	Data should be collected using standard definitions, and use of data standards for clinical trial is essential. Clinical trial data should be made available for sharing so as to conduct individual patient–level data analyses. Such databases are used by WHO and other recommending bodies for policy development. GRADE method should be well understood by all stakeholders	As data quality improves, recommendations based on lower-quality data should be reexamined. A relevant process to address this should be established.	

Abbreviations: GRADE, *Grading of Recommendations Assessment*, *Development*, *and Evaluation*; MDR, multidrug-resistant; TB, tuberculosis; WHO, World Health Organization

Drug and regimen developers already have formal mechanisms of communication with regulators, but the engagement of policy recommendation institutions should be actively encouraged and pursued as early as possible at design stages. One example of the value of such communication relates to the definition of outcomes selected for trials. Discussions with regulatory authorities usually identify endpoints that address foundations of efficacy, safety, and tolerability in studies with shorter follow-up duration; however, these outcomes may not provide adequate information for guideline developers and policy makers to endorse a given drug for use in regimens. Integration of long-term outcomes into TB trials as much as is feasible, along with the standardization of outcomes, should be a top priority for the TB therapeutics field, using, for example, the novel Phase IIC design, wherein follow-up is extended and the experimental regimens are used for their intended total duration [37].

Finally, standardized data collection and outcome definitions compatible with the Clinical Data Interchange Standards Consortium (CDISC) platforms are required by regulatory bodies. These have enhanced the ability to optimally use GRADE-based methodological approaches to evaluating the evidence, and should be similarly considered by policy makers. The application of such data standards to cohorts and the collection of national TB program data would be an invaluable step forward by allowing real-world data analyses that will greatly inform policy decisions. Until then, TB clinical trialists and regimen developers are strongly encouraged to share individual patient–level data with policy makers to permit meta-analytic data synthesis approaches to be used in the GRADE methodology [38]. Data sharing in the domain of TB is a matter of global public good, and funders, donors, and implementers of trials should not only mandate such expectations for their clinical trials but also allocate funding to support the careful curation of data accessible to the public and to policy makers for future analyses.

## Conclusion

Given the recent enthusiasm for pursuing novel trial designs in TB therapeutics [37, 39], more interactions will be needed between researchers responsible for designing the next generation of TB trials, regulators, and policy makers. This will allow better harmonization across the research pipeline and subsequent policies on access to TB medicines. Further, stakeholders, including donors and funders, need to acknowledge that both explanatory and pragmatic trials are needed to answer questions about efficacy and safety (explanatory) as well as expected effectiveness in programmatic conditions (pragmatic). In all cases, endpoints should be specific to the purposes. Late-phase clinical trial outputs that serve the objective of registration of a new TB drug or regimen can indeed meet the needs for development of public health guidelines, provided that data on long-term, patient-relevant, and population-relevant outcomes are being collected. Additionally, public health factors such as feasibility, acceptability, resource use, equity, and quality of life should be part of data collections, as these are necessary when formulating public health recommendations. The existing dialogue between drug developers and regulators should be expanded to policy makers under formal mechanisms of consultation, such as the one offered by WHO Task Forces [19]. More effective input from policy makers could greatly streamline and strengthen the value of TB clinical trial data in clinical settings. Such interactions with policy makers can be invaluable at the design stages and would result in better harmonization between the research pipeline and policies on access to TB medicines. The broad discussions that we propose would also ensure that secondary pooled analyses performed by WHO (or other policy-recommending bodies) are reliable and that the risk of conflicting interpretation and messaging provided by investigators and policy makers is reduced and usefully contribute to the generation of reliable and relevant data for further policy guidance on the treatment of all forms of TB [2].

## Abbreviations

AE: adverse event
BMRC: British Medical Research Council
CDC: Centers for Disease Control
CDISC: Clinical Data Interchange Standards Consortium
Cfz: clofazimine
CROI: Conference on Retroviruses and Opportunistic Infections
Del: delamanid; DR, drug-resistant
DS: drug-sensitive
E: ethambutol
EBA: early bactericidal activity
EMA: European Medicine Agency
FDA: Food and Drug Administration
GRADE: *Gradings of Recommendations Assessment*, *Development*, *and Evaluation*
H: isoniazid
HIV: human immunodeficiency virus
IND: investigational new drug
ITT: intention-to-treat
IUATLD: International Union Against Tuberculosis and Lung Diseases
Lfx: levofloxacin
Lzd: linezolid
M: moxifloxacin
MDR: multidrug-resistant
MGIT: mycobacterial growth in-tube
MIC: minimum inhibitory concentration
mITT: modified intent-to-treat
MSF: Médecins Sans Frontiers
NI: noninferiority
NIAID: National institute of Allergy and Infectious Diseases
NUS: National University of Singapore
OBR: optimized background regimen
orig: originally
ped: pediatric
PP: per protocol
Pret: pretomanid
R: rifampin 10 mg/kg
R35: rifampin at 35 mg/kg
Rpt: rifapentine
RR: rifampicin-resistant
TB: tuberculosis
TBTC: TB Trials Consortium
TTP: time to positivity
USAID: US Development Aid Agency
WHO: World Health Organization
Z: pyrazinamide
